# First Insights into the Phylogenetic Diversity of *Mycobacterium tuberculosis* in Nepal

**DOI:** 10.1371/journal.pone.0052297

**Published:** 2012-12-26

**Authors:** Bijaya Malla, David Stucki, Sonia Borrell, Julia Feldmann, Bhagwan Maharjan, Bhawana Shrestha, Lukas Fenner, Sebastien Gagneux

**Affiliations:** 1 Swiss Tropical and Public Health Institute (Swiss TPH), Basel, Switzerland; 2 University of Basel, Basel, Switzerland; 3 German Nepal Tuberculosis Project, Kathmandu, Nepal; 4 Institute of Social and Preventive Medicine, University of Bern, Bern, Switzerland; Institut de Génétique et Microbiologie, France

## Abstract

**Background:**

Tuberculosis (TB) is a major public health problem in Nepal. Strain variation in *Mycobacterium tuberculosis* may influence the outcome of TB infection and disease. To date, the phylogenetic diversity of *M. tuberculosis* in Nepal is unknown.

**Methods and Findings:**

We analyzed 261 *M. tuberculosis* isolates recovered from pulmonary TB patients recruited between August 2009 and August 2010 in Nepal. *M. tuberculosis* lineages were determined by single nucleotide polymorphisms (SNP) typing and spoligotyping. Drug resistance was determined by sequencing the hot spot regions of the relevant target genes. Overall, 164 (62.8%) TB patients were new, and 97 (37.2%) were previously treated. Any drug resistance was detected in 50 (19.2%) isolates, and 16 (6.1%) were multidrug-resistant. The most frequent *M. tuberculosis* lineage was Lineage 3 (CAS/Delhi) with 106 isolates (40.6%), followed by Lineage 2 (East-Asian lineage, includes Beijing genotype) with 84 isolates (32.2%), Lineage 4 (Euro-American lineage) with 41 (15.7%) isolates, and Lineage 1 (Indo-Oceanic lineage) with 30 isolates (11.5%). Based on spoligotyping, we found 45 different spoligotyping patterns that were previously described. The Beijing (83 isolates, 31.8%) and CAS spoligotype (52, 19.9%) were the dominant spoligotypes. A total of 36 (13.8%) isolates could not be assigned to any known spoligotyping pattern. Lineage 2 was associated with female sex (adjusted odds ratio [aOR] 2.58, 95% confidence interval [95% CI] 1.42–4.67, p = 0.002), and any drug resistance (aOR 2.79; 95% CI 1.43–5.45; p = 0.002). We found no evidence for an association of Lineage 2 with age or BCG vaccination status.

**Conclusions:**

We found a large genetic diversity of *M. tuberculosis* in Nepal with representation of all four major lineages. Lineages 3 and 2 were dominating. Lineage 2 was associated with clinical characteristics. This study fills an important gap on the map of the *M. tuberculosis* genetic diversity in the Asian region.

## Introduction

Tuberculosis (TB) caused by *Mycobacterium tuberculosis* remains a global health threat with an estimated nine million incident cases and 440,000 multidrug-resistant TB cases worldwide [1]. The incidence of TB was 163 per 100,000 population in 2010, and multidrug resistance (MDR) occurred in 2.9% of new cases and 11.7% of previously treated cases based on the most recent drug resistance survey in 2006 [2], [3]. In Nepal, the National Tuberculosis Control Programme adopted Directly Observed Short Course therapy (DOTS) in 1995.

*Mycobacterium tuberculosis* complex (MTBC) has a global phylogeographic population structure consisting of six main phylogenetic lineages [4]–[6]: Lineage 1 (also known as Indo-Oceanic Lineage), Lineage 2 (East-Asian Lineage, includes the Beijing genotype), Lineage 3 (Delhi/CAS), Lineage 4 (Euro-American Lineage), and Lineages 5 and 6 (*M. africanum* West African lineages 1 and 2). These lineages are associated with specific geographic regions and human populations [4], [7]–[9]. Lineage 2, for example, is most often isolated in countries in Asia and the former Soviet Union [10]. There is increasing evidence that in addition to host and environmental factors, the epidemiology of TB may also be influenced by bacterial strain variation [11]–[19]. For example, Lineage 2 (includes the Beijing genotype) has been repeatedly associated with drug resistance in a wide range of settings and countries [20]–[23], while a few studies could not find evidence for such an association [24]–[26].

There are several genotyping techniques to define the genetic diversity of *M. tuberculosis* [7], [27], [28]. Spoligotyping is a widely used genotyping technique [29], [30]. It is based on the repetitive DNA region known as the Direct Repeat (DR) locus in *M. tuberculosis* [28]. This region is characterized by series of direct repeats interspersed by short unique regions called “spacers”. However, these spacers exhibit a high rate of change, and convergent evolution can lead to identical genetic character states in phylogenetically unrelated strains [5], [31]. By contrast, genomic deletions and single nucleotide polymorphism (SNPs) evolve more slowly. Recent advances in comparative genomics have led to the development of more robust markers to study the genetic diversity [7], [32]–[36], and are therefore ideal for determining phylogenetic lineages and sub-lineages [11].

Nepal lies between two high TB burden countries, India and China which together account for one third of the world’s TB cases [37]. To date, there are no data on the phylogenetic diversity of *M. tuberculosis* in Nepal. The aims of the study were to describe the main *M. tuberculosis* lineages and spoligotypes circulating in Nepal, and to explore possible associations with clinical and epidemiological characteristics.

## Methods

### Ethics Statement

This study was approved by the Nepal Health Research Council, Nepal and the Ethics Committee of the Canton of Basel (EKBB), Switzerland. All study participants provided written informed consent. After diagnosis, the TB cases were referred to DOTS centers for treatment as provided by the Nepal Government’s National TB Control Program.

### Study Setting

The study was based on a convenience sample of TB patients mainly representing populations from Kathmandu and the surrounding area. TB suspects who reported symptoms of TB including cough for more than two weeks, chest pain, night sweat and fever were recruited at the German Nepal Tuberculosis Project (GENETUP), Kathmandu, Nepal. Patients already undergoing DOTS therapy were also enrolled, if found smear-positive during follow-up visits. GENETUP is a national reference laboratory, technically and financially supported by “Kuratorium Tuberkulose in der Welt e. V.” (Gauting, Germany), and is the main referral center for culture and drug susceptibility testing to diagnose MDR and extensively drug-resistant TB.

### Study Population and Data Collection

We included a total of 261 culture-confirmed TB cases diagnosed between August 2009 and August 2010. We collected socio-demographic and clinical data including previous TB episodes, treatment history, HIV, and BCG vaccination status. The information was collected by physicians and trained medical and nursing staff. A new case of TB was defined as a patient who had not taken anti-TB drugs for at least one month according to WHO guidelines [38]. A previously treated case was defined as a patient who received TB treatment for one month or more. BCG vaccination status was defined based on the presence or absence of a BCG scar.

### Culture, DNA Extraction and Identification of *M.* *tuberculosis* Complex

Sputum samples were cultured on Löwenstein Jensen (LJ) growth medium following standard microbiological laboratory procedures. The DNA was extracted by re-suspension of MTBC colonies in 500 µl of sterile distilled water, heat killed at 90°C for one hour, and centrifuged. The supernatants were preserved at 4°C until further use. MTBC strains were identified by multiplex polymerase chain reaction (PCR) by targeting the *rpoB* gene region. We used the forward primers K-0155 (5′-TCCTCGATGACGCCGCTTTCT-3′) and K-0209 (5′-AYATCGACCACTTCGGYAACC-3′), and the reverse primer K-0156 (5′-TCRGAGATCTTGCGCTTCTGS-3′). PCR conditions were as follows: initial denaturation step for 5 minutes at 96°C, 35 amplification cycles of 96°C for 40 secs (denaturation), 62°C for 30 secs (annealing), 72°C for 1 min (extension), and a final extension cycle of 7 minutes at 72°C. The amplicons were separated by electrophoresis on a 2% agarose gel. The PCR yielded a 849 bp amplicon in *M. tuberculosis* isolates, compared to a 1539 bp amplicon in non-tuberculous mycobacteria. All *M. tuberculosis* isolates were stored in glycerol medium at –70^o^ C.

### Determination of the Main *M. tuberculosis* Lineages

We determined the main phylogenetic lineages of *M. tuberculosis* by real-time PCR using fluorescence-labeled probes (Taqman, Applied Biosystems, USA) targeting lineage-specific SNPs as previously described [4], [32], [36].

### Spoligotyping

Spoligotyping was performed according to the manufacturer’s instructions, using commercially available kits from Isogen Bioscience BV (Maarssen, The Netherlands) [30], [39]. Spoligotyping patterns were defined according to the definitions in the SITVITWEB database (http://www.pasteur-guadeloupe.fr:8081/SITVIT_ONLINE/) accessed on October 22, 2012. The SITVITWEB global database has documented 7,105 spoligotyping patterns from a global collection of 53,816 strains [40]. All patterns that could not be assigned were considered orphan spoligotypes.

### Molecular Drug Resistance Testing

As phenotypic drug susceptibility testing results were not available for all strains, we used molecular methods to detect drug resistance in our study. Molecular drug resistance testing was performed on all strains by direct sequencing of the hotspot regions of the target genes for rifampicin (*rpoB*), isoniazid (*inhA* promoter region and *katG*), and streptomycin (*rpsL*). MDR strains were then further sequenced and analyzed for ethambutol (*embB)*, fluoroquinolones (*gyrA*) and aminoglycoside (*rrs)* resistance by sequencing of the relevant gene segments. For the *rpoB* region, we used an in-house PCR assay with primer pair K-0155 and K-0209 as described above. For all other target genes, PCR primers and PCR conditions were adapted from previously published studies [41]–[43].The sequences were analyzed with *M. tuberculosis* H37Rv as reference sequence using the Staden software package [44], [45], and compared to the publicly available web-based database (http://www.tbdreamdb.com/) [46]. Any drug resistance was defined as resistance to isoniazid, rifampicin, streptomycin, ethambutol, fluoroquinolones, and/or aminoglycosides. MDR was defined as resistance to at least isoniazid and rifampicin.

### Statistical Analyses

We used Chi-square test to test the statistical significance of differences between groups in binary variables, and the Kruskal Wallis rank test for continuous variables. Logistic regression models were used to compare patient characteristics associated with Lineage 2 (includes the Beijing genotype) compared to all other lineages (Lineages 1, 3 and 4), adjusted for age, sex, treatment history, BCG vaccination status, and any drug resistance. All statistical analyses were performed in STATA 10.1 (Stata Corp., College Station, TX, USA).

## Results

### Patient Characteristics

Of the 261 patients included in this study, 164 (62.8%) were new TB cases. Overall, 182 (69.73%) were male, and the median age was 31 years (interquartile range [IQR] 23–50). Females were significantly younger than males (median age 26 versus 35 years, p<0.001). HIV status was known in 26 patients; of these 8 (30.8%) were HIV-positive. Most patients originated from Kathmandu valley (153 cases, 58.6%), followed by 104 cases (39.8%) from different districts of Nepal, and four patients (1.5%) who were born in India.

### *Mycobacterium tuberculosis* Genotyping and Lineage Assignment

We analyzed a total of 261 *M. tuberculosis* isolates (one from each patient). The SNP-typing results showed the presence of four different *M. tuberculosis* lineages (Table 1). The most frequent lineages were Lineage 3 (includes CAS/Delhi) with 106 isolates (40.6%) and Lineage 2 (East-Asian lineage, includes Beijing genotype) with 84 isolates (32.2%). Forty one isolates (15.7%) belonged to Lineage 4 (Euro-American Lineage), and 30 isolates (11.5%) to Lineage 1 (Indo-Oceanic Lineage). Lineages 5 and 6 (*M. africanum* West African lineages) were not found in our sample.

**Table 1:** Description of the main *Mycobacterium tuberculosis* lineages and spoligotyping patterns from Nepal (total number of isolates analyzed: n = 261).

Lineage	Spoligotyping family	Spoligotyping pattern (spacers 1–43)	SIT	n	%
1	EAI5	▪▪▪▪▪▪▪▪▪▪▪▪▪▪▪▪▪▪▪▪▪▪▪▪▪▪▪▪□□□□▪□▪▪▪▪▪□□□□	138	10	33.33
1	EAI3_IND	▪□□▪▪▪▪▪▪▪▪▪▪▪▪▪▪▪▪▪▪▪▪▪▪▪▪▪□□□□▪□▪▪□□□▪▪▪▪	11	4	13.33
1	EAI1_SOM	□□□▪▪▪▪▪▪▪▪▪▪▪□▪▪▪▪▪▪▪▪▪▪▪▪▪□□□□▪□▪▪▪▪▪□▪▪▪	1734	1	3.33
1	EAI1_SOM	▪▪▪▪▪▪▪▪▪▪▪▪▪▪▪▪▪▪▪▪▪▪▪▪▪▪▪▪□□□□▪□▪▪▪▪▪□▪▪▪	48	1	3.33
1	EAI2_MANILLA	▪▪□▪▪▪▪▪▪▪▪▪▪▪▪▪▪▪▪□□▪▪▪▪▪▪▪□□□□▪□▪▪▪▪▪▪▪▪▪	19	1	3.33
1	EAI6_BGD1	▪▪▪▪▪▪▪▪▪▪▪▪▪▪▪▪▪▪▪▪▪▪□▪▪▪▪▪□□□□▪□▪▪□▪▪▪▪▪▪	292	1	3.33
1	Orphan	▪▪▪▪▪▪▪▪▪▪▪▪▪▪▪▪▪▪▪▪▪▪▪▪▪▪□□□□□□▪□▪▪▪▪▪□□□□	-	1	3.33
1	Orphan	□□□▪▪▪▪▪▪▪▪▪▪□▪▪▪▪▪▪▪▪▪▪▪▪▪▪□□□□▪□▪▪□□□▪▪▪▪	-	1	3.33
1	Orphan	▪▪▪▪▪▪▪▪▪▪▪▪▪▪▪▪▪▪▪▪▪▪▪▪▪▪□▪□□□□▪□▪▪▪▪▪□▪▪▪	-	1	3.33
1	Orphan	▪▪▪▪▪▪▪▪▪□▪▪▪▪▪▪▪▪▪▪▪▪▪▪▪▪▪▪□□□□▪□▪▪▪▪□□□□□	-	1	3.33
1	Orphan	▪▪▪▪▪▪▪▪▪▪▪▪▪▪▪▪▪▪▪▪▪▪▪▪▪▪▪▪□□□□▪□□□▪▪▪□□□□	-	1	3.33
1	Orphan	▪▪▪▪▪▪▪▪▪▪▪□▪▪▪▪▪▪▪▪▪▪▪▪▪▪▪▪□□□□▪□▪▪▪▪▪□□□□	-	1	3.33
1	Orphan	▪▪▪▪▪▪▪▪▪▪▪▪▪□□□▪▪▪▪▪▪▪▪▪▪▪▪□□□□▪□▪▪▪▪▪□□□□	-	1	3.33
1	Orphan	▪▪▪▪▪▪▪▪▪▪▪▪▪▪▪▪▪▪▪▪▪▪□▪□□□▪□□□□□□▪▪□▪▪▪▪▪▪	-	1	3.33
1	Orphan	▪▪▪▪▪▪▪▪▪▪▪▪▪□□□□▪□□□□□□□▪□□□□□□□□▪▪□▪▪▪▪▪▪	-	1	3.33
1	Orphan	▪▪▪▪▪▪▪▪▪▪▪▪▪▪□□□□▪▪▪▪▪▪▪▪▪▪□□□□▪□▪▪▪▪▪□□□□	-	1	3.33
1	Orphan	▪□□▪▪▪▪▪▪▪▪▪▪□▪▪▪▪▪▪▪▪▪▪▪▪▪▪□□□□▪□▪▪□□□▪▪▪▪	-	1	3.33
1	Orphan	▪□□▪▪▪▪□□□□□□□□□□□▪▪▪▪▪▪▪▪▪▪□□□□▪□▪▪□□□▪▪▪▪	-	1	3.33
Subtotal				30	100.00
2	Beijing	□□□□□□□□□□□□□□□□□□□□□□□□□□□□□□□□□□▪▪▪▪▪▪▪▪▪	1	82	97.62
2	Beijing	□□□□□□□□□□□□□□□□□□□□□□□□□□□□□□□□□□▪▪▪▪▪▪□▪▪	941	1	1.19
2	Beijing-like	□□□□□□□□□□□□□□□□□□□□□□□□□□□□□□□□□□□□□▪▪▪▪▪▪	250	1	1.19
Subtotal				84	100.00
3	CAS1_DELHI	▪▪▪□□□□▪▪▪▪▪▪▪▪▪▪▪▪▪▪▪□□□□□□□□□□□□▪▪▪▪▪▪▪▪▪	26	52	49.06
3	CAS	▪▪▪□□□□▪▪▪▪▪▪▪▪▪▪▪▪□□□□□□□□□□□□□□□□□▪▪▪▪▪▪▪	599	9	8.49
3	CAS	▪▪▪□□□□▪▪▪▪▪▪▪▪▪▪▪▪▪▪▪□□□□□□□□□□□□□□▪▪▪▪▪▪▪	357	6	5.66
3	CAS2	▪▪▪□□□□□□□▪▪▪▪▪▪▪▪▪▪▪▪□□□□□□□□□□□□▪▪▪▪▪▪▪▪▪	288	3	2.83
3	CAS1_DELHI	▪▪▪□□□□▪▪▪▪▪▪▪▪▪▪▪▪▪▪▪□□□□□□□□□□□□▪▪□▪▪▪▪▪▪	428	3	2.83
3	CAS1_DELHI	▪▪▪□□□□▪▪▪▪▪▪▪▪▪▪▪▪▪▪▪□□□□□□□□□□□□▪□▪▪▪▪▪▪▪	1091	3	2.83
3	CAS1_DELHI	▪▪▪□□□□▪▪▪▪▪▪▪▪▪▪▪▪▪▪▪□□□□□□□□□□□□▪▪□□□□□▪▪	2147	2	0.94
3	CAS1_DELHI	▪▪▪□□□□▪▪▪▪▪▪▪▪▪▪▪▪▪▪▪□□□□□□□□□□□□▪▪□□▪▪▪▪▪	25	1	0.94
3	U (CAS_ANCESTOR)	▪▪▪□□□□▪▪▪▪▪▪▪▪▪▪▪▪▪▪▪□□▪▪▪▪▪▪▪▪▪□□□□▪▪▪▪▪▪	27	1	0.94
3	CAS1_DELHI	▪▪▪□□□□▪▪▪▪▪▪▪□▪▪▪▪▪▪▪□□□□□□□□□□□□▪▪▪▪▪▪▪▪▪	141	1	0.94
3	CAS	▪▪▪□□□□▪▪▪▪▪▪▪▪▪▪▪▪▪▪□□□□□□□□□□□□□▪▪▪▪▪▪▪▪▪	142	1	0.94
3	CAS	▪▪▪□□□□▪▪▪▪▪▪▪▪▪▪▪▪▪▪▪□□□□□□□□□□□□□▪▪▪▪▪▪▪▪	203	1	0.94
3	CAS1_DELHI	▪▪▪□□□□▪▪▪▪▪▪▪▪▪▪▪▪▪▪▪□□□□□□□□□□□□▪▪□□□▪▪▪▪	381	1	0.94
3	CAS1_DELHI	▪▪▪□□□□▪▪▪▪▪▪▪▪▪▪▪▪▪▪▪□□□□□□□□□□□□▪▪▪▪▪□▪▪▪	429	1	0.94
3	CAS	▪▪▪□□□□▪▪▪▪▪▪▪▪▪▪▪▪▪□□□□□□□□□□□□□□▪▪▪▪▪▪▪▪▪	1093	1	0.94
3	CAS	▪▪▪□□□□▪▪▪▪▪▪▪▪▪▪▪□□□□□□□□□□□□□□□□□□▪▪▪▪▪▪▪	1422	1	0.94
3	CAS	▪▪▪□□□□□▪▪▪▪▪▪▪▪▪▪▪▪▪▪□□□□□□□□□□□□▪▪▪▪▪▪▪▪▪	1551	1	0.94
3	CAS1_DELHI	▪▪▪□□□□▪▪▪▪▪▪▪▪▪▪▪□▪▪▪□□□□□□□□□□□□▪▪▪▪▪▪▪▪▪	1590	1	0.94
3	CAS1_DELHI	▪▪▪□□□□▪▪▪▪▪▪▪▪▪▪▪▪▪▪▪□□□□□□□□□□□□□□□□▪▪▪▪▪	1789	1	0.94
3	Beijing 1	□□□□□□□□□□□□□□□□□□□□□□□□□□□□□□□□□□▪▪▪▪▪▪▪▪▪	1	1	0.94
3	Orphan	▪▪▪□□□□▪▪▪▪▪▪▪▪▪□▪▪▪▪▪□□□□□□□□□□□□□□□□▪▪▪▪▪	-	3	0.94
3	Orphan	▪▪▪□□□□▪▪▪▪▪▪▪▪▪□□□□□□□□□□□□□□□□□□□□□□□▪▪▪▪	-	1	0.94
3	Orphan	▪▪▪□□□□□□□□□□□□□□□□□□□□□□□□□□□□□□□▪▪▪▪▪□▪▪▪	-	1	0.94
3	Orphan	▪▪▪□□□□▪▪▪▪▪▪□□▪▪▪▪▪▪▪□□□□□□□□□□□□▪▪▪▪▪▪▪▪▪	-	1	0.94
3	Orphan	▪▪▪□□□□▪▪▪▪▪▪▪▪▪▪▪▪□□□□□□□▪□□□□□□□□□▪▪▪▪▪▪▪	-	1	0.94
3	Orphan	▪▪▪□□□□▪▪▪▪▪▪▪▪▪□▪▪▪▪▪□□□□□□□□□□□□▪▪▪□▪▪▪▪▪	-	1	0.94
3	Orphan	▪▪▪□□□□▪▪▪▪▪▪▪▪▪▪▪▪▪▪▪□▪▪▪▪▪▪▪▪▪▪□□□▪▪▪▪▪▪▪	-	1	1.89
3	Orphan	▪▪□□□□□▪▪▪▪▪▪▪▪▪▪▪▪▪▪▪□□□□□□□□□□□□▪▪□□▪▪▪▪▪	-	1	0.94
3	Orphan	▪▪▪□□□□▪▪▪▪▪▪▪▪□□□□□□□□□□□□□□□□□□□□□□□□□▪▪▪	-	1	0.94
3	Orphan	▪▪▪□□□□▪▪▪▪▪▪▪▪▪□▪▪▪▪▪□□□□□□□□□□□□▪▪▪□□□□□▪	-	1	0.94
3	Orphan	▪▪▪□□□□□□□▪▪▪▪▪▪▪▪▪▪▪▪□□□□□□□□□□□□□□□□□□□□□	-	1	0.94
3	Orphan	▪▪▪□□□□▪▪▪▪▪▪▪▪▪▪▪▪▪▪□□□□□□□□□□□□□□□□□□□□□□	-	1	2.83
3	Orphan	▪▪▪□□□□▪▪▪▪▪▪▪▪□□□□□□□□□□□□□□□□□□□□□□□□□▪▪▪	-	1	0.94
Subtotal				106	100.00
4	H3	▪□□▪▪▪▪▪▪▪▪▪▪▪▪▪▪▪▪▪▪▪▪▪▪▪▪▪▪▪□▪□□□□▪▪▪▪▪▪▪	655	6	14.63
4	T2	▪▪▪▪▪▪▪▪▪▪▪▪▪▪▪▪▪▪▪▪▪▪▪▪▪▪▪▪▪▪▪▪□□□□▪▪▪▪▪▪▪	52	2	4.88
4	T3	▪▪▪▪▪▪▪▪▪▪▪▪□▪▪▪▪▪▪▪▪▪▪▪▪▪▪▪▪▪▪▪□□□□▪▪▪▪▪▪▪	37	3	7.32
4	S	▪▪▪▪▪▪▪▪□□▪▪▪▪▪▪▪▪▪▪▪▪▪▪▪▪▪▪▪▪▪▪□□□□▪▪▪▪▪▪▪	34	2	4.88
4	T1	▪▪▪▪▪▪▪▪▪▪▪▪▪▪▪▪▪▪▪▪▪▪▪▪▪▪▪▪▪▪▪▪□□□□▪▪▪▪▪▪▪	53	4	9.76
4	X2	▪▪▪▪▪▪▪▪▪▪▪▪▪▪▪▪▪□▪▪▪▪▪▪▪▪▪▪▪▪▪▪□□□□▪▪□□□□▪	137	2	4.88
4	T1	▪▪▪▪▪▪▪▪▪▪▪▪▪▪▪▪▪▪▪▪▪▪▪▪▪▪▪▪▪▪▪▪□□□□▪▪□□□□▪	244	2	4.88
4	H1	▪▪▪▪▪▪▪▪▪▪▪▪▪▪▪▪▪▪▪▪▪▪□□▪□□□□□□▪□□□□▪▪▪▪▪▪▪	620	2	4.88
4	LAM9	▪▪▪▪▪▪▪▪▪▪▪▪▪▪▪▪▪▪▪▪□□□□▪▪▪▪▪▪▪▪□□□□▪▪▪▪▪▪▪	42	1	2.44
4	H3	▪▪▪▪▪▪▪▪▪▪▪▪▪▪▪▪▪▪▪▪▪▪▪▪▪▪▪▪▪▪□▪□□□□▪▪▪▪▪▪▪	50	1	2.44
4	T2–T3	▪▪▪▪▪▪▪▪▪▪▪▪□▪▪▪▪▪▪▪▪▪▪▪▪▪▪▪▪▪▪▪□□□□▪▪▪□▪▪▪	73	1	2.44
4	T1	▪▪▪▪□▪▪▪▪▪▪▪▪▪▪▪▪▪▪▪▪▪▪▪▪▪▪▪▪▪▪▪□□□□▪▪▪▪▪▪▪	154	1	2.44
4	T1	▪▪▪▪▪▪▪▪▪▪▪▪□□□□▪▪▪▪▪▪▪▪▪▪▪▪▪▪▪▪□□□□▪▪▪▪▪▪▪	102	1	2.44
4	X1	▪▪▪▪▪▪▪▪▪▪▪▪▪▪▪▪▪□▪▪▪▪▪▪▪▪▪▪▪▪▪▪□□□□▪▪▪▪▪▪▪	119	1	2.44
4	H1	▪▪▪▪▪▪▪▪▪▪▪▪▪▪▪▪▪▪▪▪▪□□□▪□□□□□□▪□□□□▪▪▪▪▪▪▪	283	1	2.44
4	T1	▪▪▪▪▪▪▪▪▪▪▪▪▪▪▪▪▪▪▪▪▪▪▪▪▪▪▪▪▪▪▪▪□□□□▪▪▪▪▪□□	628	1	2.44
4	H3	▪▪▪▪▪▪▪▪▪▪▪▪□▪▪▪▪▪▪▪▪▪▪▪▪▪▪▪▪□□▪□□□□▪▪▪▪▪▪▪	929	1	2.44
4	Orphan	▪▪▪▪▪▪▪▪▪▪▪▪▪▪▪▪▪▪▪▪□▪□□□□▪▪▪▪▪▪□□□□▪▪▪▪▪▪▪	-	1	2.44
4	Orphan	□▪▪▪▪▪□▪▪▪▪▪▪▪▪▪▪□▪▪▪▪▪▪▪▪▪▪▪▪▪▪□□□□▪▪▪▪▪▪▪	-	1	2.44
4	Orphan	▪▪▪▪▪▪□▪▪□▪▪▪▪▪▪▪▪□▪▪▪▪▪▪▪▪▪▪▪▪▪□□□□□▪▪▪▪▪▪	-	1	2.44
4	Orphan	▪□▪▪▪▪▪▪▪▪▪▪▪▪□□▪▪▪▪▪▪▪▪▪▪▪▪▪▪▪▪□□□□▪▪▪▪▪▪▪	-	1	2.44
4	Orphan	▪□▪▪▪▪▪▪▪▪▪▪▪▪▪▪▪▪▪▪▪▪▪▪▪▪▪▪▪▪▪▪□□□□□▪▪▪▪▪▪	-	1	2.44
4	Orphan	▪▪▪□▪▪▪▪□□▪▪□▪▪▪▪▪▪▪▪▪▪▪▪▪▪▪▪▪▪▪□□□□▪▪▪▪□▪▪	-	1	2.44
4	Orphan	▪▪▪▪▪▪▪▪▪▪▪▪▪▪▪▪▪▪▪▪□▪▪□□□□□□□□▪□□□□▪▪▪▪▪▪▪	-	1	2.44
4	Orphan	▪▪▪▪▪□▪▪▪▪▪▪▪▪▪▪▪▪□▪▪▪▪▪▪▪▪▪▪▪▪▪□□□□▪▪▪▪▪▪▪	-	1	2.44
4	Orphan	▪▪□▪▪▪▪▪▪▪▪▪▪▪▪▪▪▪▪▪▪▪▪▪▪▪□□□□□□□□□□▪▪▪▪▪▪▪	-	1	2.44
Subtotal				41	100.00

SIT, Spoligotype International Type according to the definitions in the SITVITWEB database (http://www.pasteur-guadeloupe.fr:8081/SITVIT_ONLINE/) accessed on April 2012.

1 This strain was assigned to Lineage 3 (Delhi/CAS) based on alternative molecular markers, as previously published [31].

Lineage 1: Indo-Oceanic Lineage; Lineage 2: East-Asian Lineage (includes Beijing strains); Lineage 3: Delhi/CAS; Lineage 4: Euro-American Lineage.

Based on spoligotyping, we detected 45 different spoligotypes (SITs) corresponding to 225 *M. tuberculosis* isolates (Table 1). The remaining 36 (13.8%) strains could not be assigned to any known spoligotyping pattern in the SITVITWEB database, and were therefore considered orphan spoligotypes. The spoligotyping results showed that CAS family (90, 34.5%) and Beijing (84 isolates, 32.2%) were the predominant spoligotypes in our sample (Table 1). Among the CAS family, the most prevalent spoligotype was CAS1_DELHI (SIT 26) representing 52 (19.9%) isolates, and almost all Beijing isolates (83 of 84 isolates belonging to Lineage 2) showed the classical Beijing spoligotyping pattern. Of the 41 strains belonging to Lineage 4, we found spoligotypes that have been reported before in India or Tibet (LAM9, H3, T2–T3, T1, XI, H1, and H3) according to the SITVITWEB database. Among the 30 (11.5%) Lineage 1 strains, only 18 (60.0%) matched the SITs of the East African Indian (EAI) family. Only two SIT types SIT 138 (EAI5; n = 10), and SIT 11 (EAI3_IND; n = 4) were represented by more than one strain. However, SIT 1734 (EAI1_SOM) present as a single isolate in our dataset was not reported before from the Indian sub-continent according to the SITVITWEB database.

When comparing SNP typing with the spoligotyping results, we found one case of “pseudo-Beijing” spoligotpype as previously reported (Table 1) [31].

### Drug Resistance

Overall, 50 (19.2%) *M. tuberculosis* isolates had any drug resistance, and 16 (6.1%) were MDR as determined by DNA sequencing of the main target regions (Table 2). Any drug resistance was more frequently detected among previously treated TB cases (29 cases, 30.0%) compared to new cases (21 cases, 12.8%, p = 0.001). Among the 16 MDR strains, 9 (56.3%) were assigned to Lineage 2 (East-Asian Lineage), 6 (37.5%) to Lineage 3 (CAS/Delhi), and one (6.2%) to Lineage 4 (Euro-American Lineage).

**Table 2:** Associations of patient characteristics across the four main *Mycobacterium tuberculosis* lineages identified in Nepal.

Patient characteristics	Total	Lineage 1	Lineage 2	Lineage 3	Lineage 4	*P* value
	n (%)	(n = 30)	(n = 84)	(n = 106)	(n = 41)	
Age, median (IQR), years	31 (23–50)	42 (24–50)	30 (23.5–50.5)	30 (23–45)	38 (23–55)	0.50
Female sex	79 (30.3)	4 (13.3)	35 (41.7)	29 (27.4)	11 (26.8)	0.016
Previously treated	97 (37.2)	8 (26.7)	39 (46.4)	35 (33.0)	15 (36.6)	0.15
BCG vaccinated	110 (42.2)	13 (43.3)	31 (36.9)	46 (43.4)	20 (48.8)	0.62
Any resistance	50 (19.2)	4 (13.3)	26 (30.9)	14 (13.2)	6 (14.6)	0.011
MDR	16 (6.1)	0	9 (10.7)	6 (5.7)	1 (2.4)	0.14 1

BCG, Bacille Calmette Guerin; IQR, Interquartile range; MDR, Multidrug-resistant.

1 Fisher’s exact test.

### Association between *M. tuberculosis* Lineages and Patient Characteristics

We observed that the proportion of female sex was different across the four main *M. tuberculosis* lineages. Lineage 2 isolates were more common among females (41.7%), compared to other lineages (range 13.3% to 27.4%, overall p = 0.016, Table 2). Moreover, any drug resistance was more frequently detected in Lineage 2 isolates (31.0%) than in any other lineages (range 13.2% to 14.6%, overall p = 0.011). Other patient characteristics such as age, previous treatment history, or BCG vaccination were not significantly associated with any of the four lineages (Table 2).

Because Lineage 2 (includes Beijing genotype) has been previously associated with particular characteristics [17], [19], [21], [47], and because Lineage 2 was the second most common lineage in our sample, we tested whether these characteristics were also associated with Lineage 2 in our setting by comparing our Lineage 2 isolates to the other lineages combined (Table 3). Logistic regression analyses showed that Lineage 2 was associated with female sex (adjusted odds ratio [aOR] 2.58; 95% confidence interval [95%CI] 1.42–4.67, p = 0.002) and any drug resistance (aOR 2.79; 95%CI 1.43–5.45, p = 0.002). A history of previous TB treatment tended to be associated with Lineage 2 (aOR 1.68, 95% CI 0.95–2.97, p = 0.074), while BCG vaccination status was not associated with Lineage 2 (includes Beijing genotype) compared to other lineages (aOR 0.67; 95%CI 0.37–1.20, p = 0.18).

**Table 3:** Multivariate associations between patient characteristics and *Mycobacterium tuberculosis* Lineage 2 (n = 84, includes the Beijing genotype) compared to all other lineages (n = 177).

Patient characteristics	Lineage 2	Unadjusted		Adjusted	
	n (%)	OR (95% CI)	*P* value	OR (95%CI)	*P* value
Age, median (IQR), years	31 (23–50)	0.99 (0.98–1.01)	0.80	0.99 (0.98–1.01)	0.99
Female sex	35 (44.30)	2.15 (1.24–3.74)	0.006	2.58 (1.42–4.67)	0.002
Previously treated	39 (40.20)	1.77 (1.04–3.02)	0.034	1.68 (0.95–2.97)	0.074
BCG vaccinated	31 (28.18)	0.72 (0.42–1.23)	0.23	0.67 (0.37–1.20)	0.18
Any resistance	26 (52.00)	2.85 (1.51–5.37)	0.001	2.79 (1.43–5.45)	0.002

BCG, Bacille Calmette Guerin; IQR, Interquartile range; OR, Odds ratio; 95% CI, 95% confidence interval.

Model was adjusted for age, sex, previous TB treatment, BCG vaccination, and any resistance.

Lineage 1, 3 and 4 were used as the comparison group.

## Discussion

We analyzed 261 *M. tuberculosis* isolates from Nepal using SNP typing and spoligotyping. We found that four main phylogenetic lineages of *M. tuberculosis* were present in Nepal. Lineage 2 (East-Asian Lineage, includes the Beijing genotype) and Lineage 3 (CAS/Delhi) were the most frequent, while Lineage 1 (Indo-Oceanic Lineage) and Lineage 4 were less prevalent. Spoligotyping revealed a large genetic diversity with the predominant spoligotyping families being Beijing and CAS/Delhi, and nearly 14% of spoligotyping patterns previously unreported.

Because Nepal is geographically located between India and Tibet (China), we expected to observe similar *M. tuberculosis* genotypes in Nepal as in these neighboring countries. Indeed, Lineage 3 (corresponds to Delhi/CAS spoligotype), which was the most common *M. tuberculosis* genotype in our sample, was previously shown to be predominant in Northern India [48]–[50]. Similarly, Lineage 2 (includes Beijing), which was the second most common genotype in our study has been reported as the most frequent among TB cases from China (including Tibet) [22], [51]–[55]. The prevalence of the Beijing genotype of 32.2% in our study is in the range of the prevalence reported from other Asian countries, ranging from 17% in Malaysia to 72% in Japan [56]. Lineage 1 which is association with South-Indian region, Bangladesh and the Philippines was also present in our study sample [4].

We observed a discrepancy between SNP typing and spoligotyping results. Spoligotyping is based on the highly variable DR locus, and convergent evolution may therefore lead to homoplasy in spoligotyping patterns [5]. We found a strain with a Beijing spoligotype, which was assigned to Lineage 3 (includes CAS genotype) rather than to Lineage 2 (includes Beijing) based on alternative molecular markers. We have previously published this phenomenon as “Pseudo-Beijing” [31]. In Asian countries with a high prevalence of Beijing spoligotypes, it is likely that this phenomenon may be observed in other settings.

We found that Lineage 2 was associated with female sex, which is in line with a previous study from Vietnam [57]. In contrast to other studies [57], [58] however, we found no evidence for an association between Lineage 2 and age. Our observation may be explained by bacterial factors or genetic host factors. Young and middle-aged women may be more likely to progress from infection to disease than men [59], [60]. Alternatively, our results may be influenced by recruitment of more young females than young males into our study. Indeed, females were younger than males in our study population. Overall, our study population showed a male-to-female ratio of 2.3∶1 which is similar to the global estimate of 1.9∶1 reported by WHO [2], and may reflect differences in access to health care [61], [62]. Furthermore, sex differences in TB case notification rates among males and females have been noted before in other settings [63], [64].

Lineage 2 was also associated with any drug resistance. This is consistent with previous studies from different settings [21], [23]. The reasons for this association remain unknown [20], but the strain genetic background of Beijing strains [65] and their interactions with the human immune system may play a role [21]. Alternatively, this association might reflect higher relapse rates in patients infected with Beijing strains [66]. Indeed, in our study, Lineage 2 included more patients that were previously treated but this association was not statistically significant. Finally, previous studies hypothesized that Beijing strain may escape the protective immunity of BCG vaccination [21], but we found no evidence for such an association between Lineage 2 and BCG immunization. BCG immunization has been introduced in Nepal more than 30 years ago, with an estimated immunization coverage of 96% in 2009 [67]. However, larger studies may be required for a more complete understanding of the association between previous BCG vaccination and particular *M. tuberculosis* genotypes.

Our study has several limitations. First, the study was not population-based as patients were recruited only at GENETUP (Kathmandu), and patients diagnosed at other microscopy centers during the study period could not be included. Second, patients coming from more remote areas outside of Kathmandu might be more likely to be referred as drug resistance suspects. Therefore, this may have artificially increased the proportion of drug-resistant strains in our sample. Third, although our study covered samples from forty different districts of Nepal including those bordering with India and Tibet, half of patients were from the Kathmandu area. Therefore, the study results mainly reflect the genetic diversity of the strains from the patients who visited GENETUP.

In conclusion, we found a high diversity of *M. tuberculosis* genotypes in Nepal with representation of all four main *M. tuberculosis* lineages, and showed that Lineage 2 (includes Beijing genotype) was associated with female sex and any drug resistance. This study fills the gap on the map of the genetic population structure of *M. tuberculosis* in the Asian region by providing a first insight into the phylogenetic lineages of *M. tuberculosis* circulating in Nepal.
